# Optimality in DNA repair

**DOI:** 10.1016/j.jtbi.2011.08.024

**Published:** 2012-01-07

**Authors:** Morgiane Richard, Matthew Fryett, Samantha Miller, Ian Booth, Celso Grebogi, Alessandro Moura

**Affiliations:** a Institute of Complex Systems and Mathematical Biology, King's College, University of Aberdeen, Aberdeen AB24 3UE, UK; b Institute of Medical Sciences, College of Life Sciences and Medicine, University of Aberdeen, Aberdeen AB25 2ZD, UK

**Keywords:** DNA damage, DNA repair, Stochastic modelling

## Abstract

DNA within cells is subject to damage from various sources. Organisms have evolved a number of mechanisms to repair DNA damage. The activity of repair enzymes carries its own risk, however, because the repair of two nearby lesions may lead to the breakup of DNA and result in cell death. We propose a mathematical theory of the damage and repair process in the important scenario where lesions are caused in bursts. We use this model to show that there is an optimum level of repair enzymes within cells which optimises the cell's response to damage. This optimal level is explained as the best trade-off between fast repair and a low probability of causing double-stranded breaks. We derive our results analytically and test them using stochastic simulations, and compare our predictions with current biological knowledge.

## Highlights

► We formulate a model for DNA damage and repair. ► Increasing the level of repair enzymes can have detrimental effects to cells. ► There is an optimum level of repair enzymes which optimises the cell's response to damage.

## 1 Introduction

Many common phenomena in Nature can lead to damage of the genetic material inside living cells, including UV light, radioactivity, reactive chemical species with high affinity for DNA, and highly reactive oxygen species generated in various ways in the intracellular medium (Sancar, 1996; Sancar et al., 2004). On the one hand, high-energy particles from radioactive sources can damage two neighbouring phosphodiester bonds which make up the DNA backbone (Steel, 1993) — a *bi-stranded damage cluster* — resulting in a potentially fatal structural damage known as *double-strand break* (DSB) which breaks the DNA molecule apart (Krasin and Hutchinson, 1977; Pennington and Rosenberg, 2007). On the other hand, when the energy of the damaging agent is low, it does not lead directly to the breakup of the DNA backbone. In this latter case, damage consists of the formation of anomalous chemical bonds in the affected nucleotides. UV photons, for example, typically induce the formation of *pyrimidine dimers*, where two neighbouring nucleotides become linked (Sancar, 1996). Even though low-energy lesions do not lead directly to the destruction of the DNA molecule, they hinder crucial cellular processes such as DNA replication and transcription. We will focus on low-energy lesions for the remainder of this paper.

**Fig. 1:**
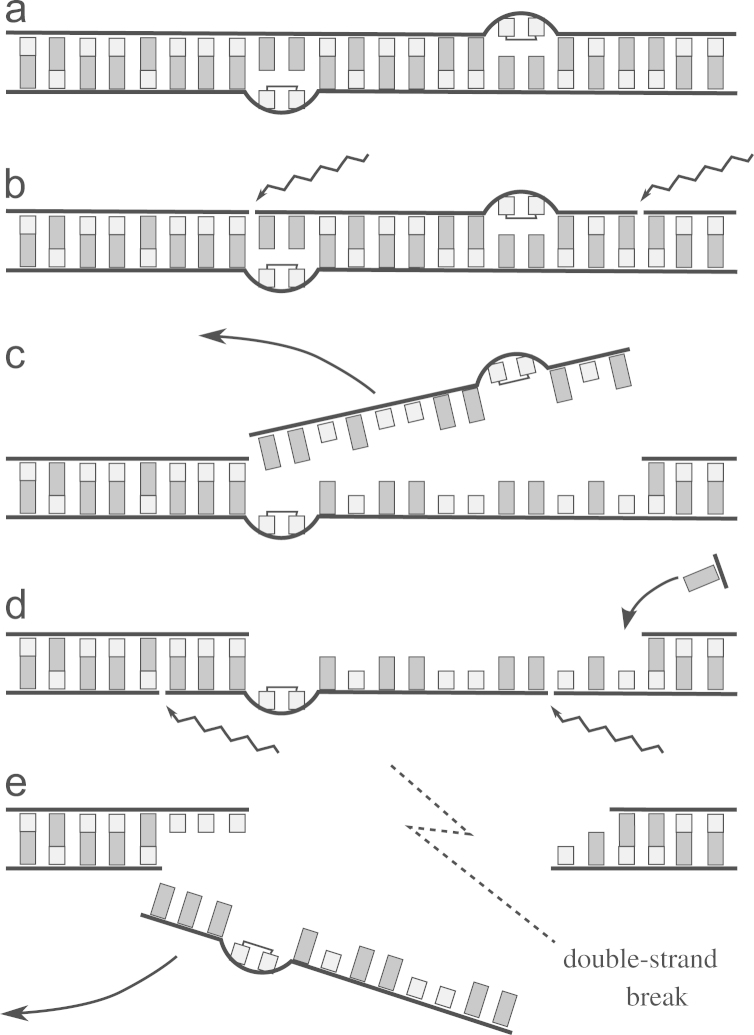
Double-strand break caused by repair of two close damage lesions. (a) A DNA molecule with two close damage sites lying on opposite strands. Repair is initiated on one of the strands, first by cutting the DNA backbone around the damage (b), followed by excision of the DNA segment with the damage (c). The excised region starts to be reconstructed using the other strand as a template (d). However, if a new repair starts in the other damaged site before the gap created by the first repair has been closed (d), a portion of the DNA molecule loses both strands and the molecule is broken into two, creating a double-strand break (e).

Cells have a number of mechanisms to repair low-energy DNA damage. One of the most important is *nucleotide excision repair* (NER) (Sancar, 1994, 1996), illustrated in Fig. 1. NER is the primary mechanism protecting cells against UV-induced damage, and will be the focus of our study, although our theory and our results are also applicable to other repair mechanisms which work in similar ways, such as *base excision repair* (Sancar et al., 2004). As depicted in Fig. 1, NER works by first recognising the presence of a damaged base, excising a piece of the DNA strand containing the damaged bases, and then rebuilding the resulting gap using the complementary strand as a template (Sancar, 1996). If all goes well, the result of this process is a new undamaged DNA molecule with a sequence identical to that of the original one.

During the repair process, while the gap left by the excision is being rebuilt, the DNA has only a single strand in the region of repair. This opens the possibility that the repair of two close lesions can cause two overlapping gaps, leading to a DSB (Harm, 1968; Moss and Davies, 1974); this is illustrated in Fig. 1. If DNA is present in more than one copy, for instance if DNA was being replicated when the damage occurs, DSBs can be fixed by the mechanism of homologous repair (Krasin and Hutchinson, 1977; Wyman and Kanaar, 2006). However, this repair mechanism is not available in slow-growing cells with limited access to nutrients, and DSBs are then usually fatal. Another repair mechanism is non-homologous end-joining repair, which does not depend on the presence of multiple copies of the DNA molecule; but this mechanism is not present in many bacteria, including *Escherichia coli*, the model organism we will focus on for the remainder of this paper.

Thus, repair itself can lead to death, which suggests that the relation between repair and the death rate can be counter-intuitive, requiring a solid mathematical formulation to be properly understood. Most works in the literature addressing the modelling of low-energy DNA damage and repair are concerned with the dynamics of the detailed biochemical events involved in DNA repair (Politi et al., 2005; Kesseler et al., 2007; Shimoni et al., 2009), including adaptation mechanisms such as the SOS response in bacteria (Aksenov et al., 1997; Ni et al., 2007); homologous repair has also been studied (Mouri et al., 2009). The direct relation of repair and death is addressed to a limited extent in Moss and Davies (1974), where the overlapping of excised DNA regions as a cause of death is explicitly taken into account. But their formulation is static, taking a given distribution of excised gaps as an input, without taking into account how they are dynamically created by the action of the repair enzymes. A recent paper took this effect into account in the case of chemically induced DNA damage in *E. coli* bacteria (Karschau et al., 2011), and revealed that the interplay between repair and death leads to surprising consequences to the dependence of mortality rates on the concentration of the damage-inducing chemical and on the number of repair enzymes present in the cell. For example, it was shown in Karschau et al. (2011) that higher numbers of repair enzymes can lead to a greater death rate.

In this paper we study a mathematical model of the repair mechanism, which takes into account the fact that repair itself is the cause of DSBs, as depicted in Fig. 1. We focus here on the case where DNA damage is generated in short, concentrated bursts, which then have to be repaired by the cells. This is a realistic scenario, corresponding for example to organisms being exposed to direct sunlight for short intervals, while staying in the shadow most of the time. After being exposed to damage, some cells in a population will recover after their DNA is repaired, and some will die because of the DSBs generated during the repair process. This transient repair dynamics is very different from the dynamics investigated in Karschau et al. (2011), where damage is continuously being created by a toxic chemical, and all cells in a population will eventually die. In the present work, since the source of damage is localised in time, the repair process takes a finite time, and so it makes sense to define a survival probability in this case, and ask how it depends on the amount of damage and on the number of repair enzymes. This formulation allows us to ask what is the optimal number of repair enzymes in a given cell: many repair enzymes result in fast repair times, but increase the probability of creating DSBs and killing the cell; on the other hand, low levels of repair enzymes mean that repair may take too long.

We will show analytically and numerically that the probability of recovering from the damage and surviving repair is a *decreasing* function of the number of repair enzymes *N*_*E*_, a result which bears some resemblance to the one reported in Karschau et al. (2011). This result simply stems from the fact that more repair enzymes make it more likely for DSBs to be created, and therefore increases the probability of DSBs being created (see Fig. 1). This may seem a paradoxical result, since it predicts that the more repair activity there is, the more likely cells are to eventually die, and we would expect repair to be beneficial to the cells. However, we will show that the average time it takes to repair all lesions decreases with *N*_*E*_, and those cells lucky enough to survive will resume their normal life more quickly. There is thus a competition between two effects: increasing *N*_*E*_ increases the probability of repair-induced DSBs, but it decreases the time of repair, allowing surviving cells to resume normal growth sooner. We show in this paper that there is a maximum value of *N*_*E*_ above which the death rate caused by DSBs generated during repair is so high that overall growth becomes negative. We also show that there is an optimum number of repair enzymes for which there is an optimum balance between the benefit of decreasing the repair time and the disadvantage of killing cells during repair. This optimum number turns out to be about 100 molecules, which is compatible with the number of repair enzymes estimated for bacterial cells. This suggests that evolution has selected this level of repair enzymes in order to maximise growth and survival.

## 2 Model definition

We assume that at an initial time, a short burst of UV light (for example) damages *N*_0_ sites on a cell's DNA, and that these *N*_0_ sites are homogeneously distributed within the chromosome. A few minutes in sunlight can result in thousands of DNA lesions in a single cell, so we regard *N*_0_ as large, but still much smaller than the DNA size *N*, which is of order 10^6^ in bacteria. The other important parameter in the system is the number of repair enzymes *N*_*E*_. In reality there are many enzymes involved in the repair process, but each individual repair is initiated by a specific enzyme, which starts the whole process. In the case of bacteria, for example, this enzyme is UvrA. So *N*_*E*_ is the number of “initiator” repair enzymes. When exposed to sudden intense DNA damage-causing agents, cells can respond by increasing the expression of genes producing repair enzymes, for example through the SOS response mechanism in bacteria (Sancar, 1996); we will assume that this takes place in a burst at the start of the repair, and that *N*_*E*_ remains constant afterwards. One important characteristic of the repair system is that each repair enzyme can only repair one site at a time. We take this into account in the model through the relation

(1) $$n_{R}+n_{E}=N_{E},$$

where *n*_*R*_ is the number of repairs currently in progress, and *n*_*E*_ is the number of free repair enzymes, available for new repairs.

The number of damaged bases *n*_*D*_ that are not currently under repair satisfies the equation

(2) $$\frac{{\mathrm{dn}}_{D}}{\mathrm{dt}}=-\alpha n_{D}n_{E}=-\alpha n_{D}(N_{E}-n_{R}),$$

where α is the rate constant for the binding of a repair enzyme to a damaged base, and we used Eq. (1). The equation for the number of sites under repair is given by

(3) $$\frac{{\mathrm{dn}}_{R}}{\mathrm{dt}}=\alpha n_{D}(N_{E}-n_{R})-\gamma n_{R},$$

where γ is the repair completion rate.

## 3 Results

### 3.1 Repair time and survival probability

Since $n_{D}âª¢N_{E}$ for most of the repair process, a time-scale analysis of Eqs. (2) and (3) shows that *n*_*R*_ reaches a quasi-steady-state very quickly, and we can consider ${\mathrm{dn}}_{R}/\mathrm{dt}\approx 0$. Substituting this in Eqs. (3) and (2) we get

(4) $$n_{R}(t)=\frac{N_{E}n_{D}}{\frac{\gamma }{\alpha }+n_{D}},\;\frac{{\mathrm{dn}}_{D}}{\mathrm{dt}}=-\frac{\gamma N_{E}n_{D}}{\frac{\gamma }{\alpha }+n_{D}}.$$

For bacterial cells, γ is estimated to be $10^{-2}\;s^{-1}$ (Husain et al., 1985; Ni et al., 2007). The value of α is harder to quantify, but it is expected to be of the same order of magnitude as γ. We can therefore assume $n_{D}âª¢\gamma /\alpha$. In this case we have

(5) $$n_{R}=N_{E},\;n_{D}(t)=N_{0}-\gamma N_{E}t.$$

Consequently, the conditions $n_{D}(t)âª¢\gamma /\alpha$ imply that $n_{D}(t)âª¢N_{E}$, so that the equilibrium value of *n*_*r*_ is *N*_*E*_. This means that the repair enzymes are maximally used. From the above equation we can find the *repair time* τ, that is, the time at which all damaged sites have been repaired (assuming for the moment that no DSB has been formed):

(6) $$n_{D}(\tau )=0\Rightarrow \tau =\frac{N_{0}}{\gamma \cdot N_{E}}.$$

τ is the average time it takes for all the *N*_0_ lesions to be repaired in a given cell, if that cell survives the repair process; from the above expression, it is inversely proportional to *N*_*E*_.

To calculate the probability that cells die as a result of repair, we need to find the probability $P_{\mathrm{DSB}}$ that any new repair starts at a position too close to another ongoing repair, leading to a DSB (see Fig. 1). Consider the chromosome at some time *t*. From Eq. (5), there are $\approx N_{E}$ repairs being carried out, in positions randomly distributed on the DNA. The critical distance *L* between ongoing repairs such that the DSB created is of the order of 10 bases. So each repair defines a “danger zone” around it of size 2L, and thus the total length of the danger zones is $2{\mathrm{LN}}_{E}$ (assuming no overlapping of danger zones, which is reasonable since $N_{E}âª¡N$). The probability of any new repair giving rise to a DSB is therefore

(7) $$P_{\mathrm{DSB}}={\mathrm{LN}}_{E}/N.$$

The factor of 2 disappears because two lesions must be on opposite strands to be able to generate a DSB.

Each new repair thus has a probability $P_{\mathrm{DSB}}$ of creating a DSB. We will assume that a constant fraction *f* of DSBs lead to cellular death, with the remaining 1−f fraction being those cells which manage to fix their DSBs somehow—for example by homologous repair. In cells where homologous recombination is not possible, such as bacteria in a limited medium, *f* is expected to be close to 1. The probability of a new repair killing the cell is therefore ${\mathrm{fP}}_{\mathrm{DSB}}$. So the probability *P* that at the end of *N*_0_ repairs the cell has not been killed (the *survival probability*) is

(8) $$P={\left(1-{\mathrm{fP}}_{\mathrm{DSB}}\right)}^{N_{0}}\approx \exp(-{\mathrm{fLN}}_{E}N_{0}/N).$$

This shows that *P* decreases with the number of repair enzymes. This is a direct consequence of the fact that more repair enzymes mean an increased probability of generating a DSB. For low doses (small *N*_0_, but with $N_{0}âª¢N_{E}$), the death probability 1−P depends linearly on the dose (*N*_0_) and on *N*_*E*_.

**Fig. 2:**
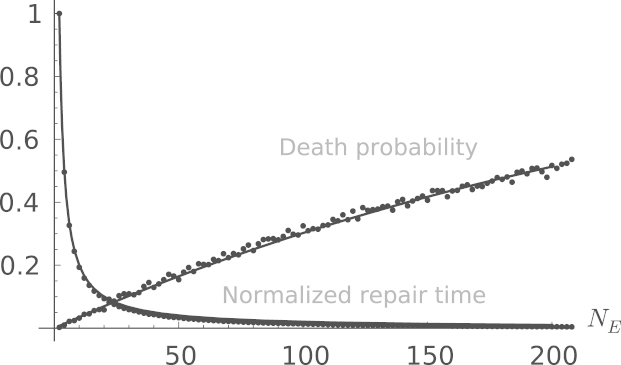
The probability 1−P that a cell will die and the average time τ it takes to repair a cell, as a function of *N*_*E*_. The repair time $\tau (N_{E})$ is normalised by $\tau (N_{E}=2)$. Full lines are analytical predictions, and dots are the results of stochastic simulations. The parameters are $\alpha =\gamma =0.01\;s^{-1}$, $N_{0}=2000$, $N=5\times 10^{6}$, *L*=9, which are reasonable for *E. coli*. We use *f*=1 throughout. We have verified that these results are independent of α, as predicted by Eqs. (6) and (8).

UV light produces about six pyrimidine dimers per 10^6^ nucleotides for a dose of 1 J/m^2^ (Rupp and Howard-Flanders, 1968; Ni et al., 2007). The intensity of UV light in a bright day is of the order of 2 W/m^2^, corresponding to 60 lesions per second being generated in an *E. coli* cell with a genome 5×10^6^ nucleotides long. So 5 s in direct sunlight would result in $N_{0}\approx 300$ dimers. Using this value for *N*_0_ as an example, and parameters appropriate for *E. coli* cells (see Fig. 2), we find a repair time τ≈25min for *N*_*E*_=20, which matches well with the observation that *E. coli* bacteria take about 20 min to recover from a 10 J/m^2^ UV irradiation before resuming normal activities (Doudney, 1990). Comparison of our predicted survival rate *P* with observations (Moss and Davies, 1974; Bronk and Walbridge, 1980) reveals that our theory tends to underestimate the death rate, suggesting that there are spatial effects which tend to increase the probability of new repairs starting close to ongoing repair sites. This supports the hypothesis put forth in Moss and Davies (1974).

### 3.2 Stochastic simulations

Since *N*_*E*_ is small in cells, one may question the validity of using differential equations as we did in our derivation above. To see if our results are preserved in the presence of the stochastic variations caused by low-abundant species such as the repair enzymes, we implement a stochastic simulation of the repair process. We used a variation of the Gillespie (2007) algorithm to simulate the random discrete changes in *n*_*D*_ and *n*_*R*_ corresponding to the continuous approximation given by Eqs. (2) and (3). In contrast to the usual Gillespie algorithm, however, we keep track of where each damaged and under-repair site is in the chromosome, so that we can tell when a DSB is created by two nearby repairs. Running many realisations of this stochastic process, we get from the simulation the repair time $\tau (N_{E})$ and the survival probability $P(n_{E})$ as a function of the number of repair enzymes. The result is plotted in Fig. 2, along with the analytical predictions given by Eqs. (6) and (8). The simulations and the analytical results match extremely well, showing that our ODE theory is valid. The parameters we used in Fig. 2 are realistic for *E. coli* cells.

## 4 Discussion

### 4.1 The optimal balance between repair and death

The above results show that repair enzymes are a double-edged sword. If *N*_*E*_ is too large, cells are very likely to die after DNA damage as a result of the repair process, as P→0 in Eq. (8). If, on the other hand, *N*_*E*_ is too low, then the repair time τ becomes very large, as seen from Eq. (6). We now derive by a simple argument the optimum value of *N*_*E*_, which achieves the best balance for cells, and we explore limitations on the number of enzymes in cells imposed by the repair dynamics.

Cells with unrepaired damage to their DNA usually cannot proceed with their normal life cycle, thus preventing them from replicating. This fact suggests there is an optimum balance between a high death rate and a long repair time. Let *T*_0_ be the normal replication time (doubling time) of a microorganism, in the absence of any DNA damage. In the simplest model, the replication time *T* of a cell subject to DNA damage increases by the time τ it takes to repair the damage: $T=T_{0}+N_{0}/(\gamma N_{E})$ (using Eq. (6)). This is consistent with experimental growth measurements in bacteria subjected to UV irradiation (Doudney, 1990). We are assuming in this idealised model of population growth that each generation of cells is exposed to a burst of radiation which causes *N*_0_ damages. So in the absence of DNA damage, the population size would be described by an exponential growth factor $\exp(\lambda_{0}t)$, with $\lambda_{0}=(\ln\;2)/T_{0}$. The presence of DNA damage slows this down to λ=(ln2)/T. In addition, damage causes cells to die at a rate such that after the time *T*, a fraction 1−P of all cells die, where *P* is given by Eq. (8). This corresponds to a death rate of μ=−(lnP)/T. The overall growth rate *R* of the population is thus

(9) $$R=\lambda -\mu =\frac{\ln\;2-{\mathrm{fLN}}_{E}N_{0}/N}{T_{0}+N_{0}/(\gamma N_{E})}.$$

Negative values of *R* would imply a decreasing population, which means these values are avoided by evolution. We therefore must have R>0, or from the above expression,

(10) $$N_{E}<N_{\max}=\frac{N\;\ln\;2}{{\mathrm{fLN}}_{0}}.$$

Thus we show that there is a maximum number $N_{\max}$ of repair enzymes beyond which the rate of death caused by DNA damage and repair exceeds the reproduction rate of those that do survive the repair process. Notice that $N_{\max}$ is inversely proportional to the initial number of damaged bases *N*_0_.

**Fig. 3:**
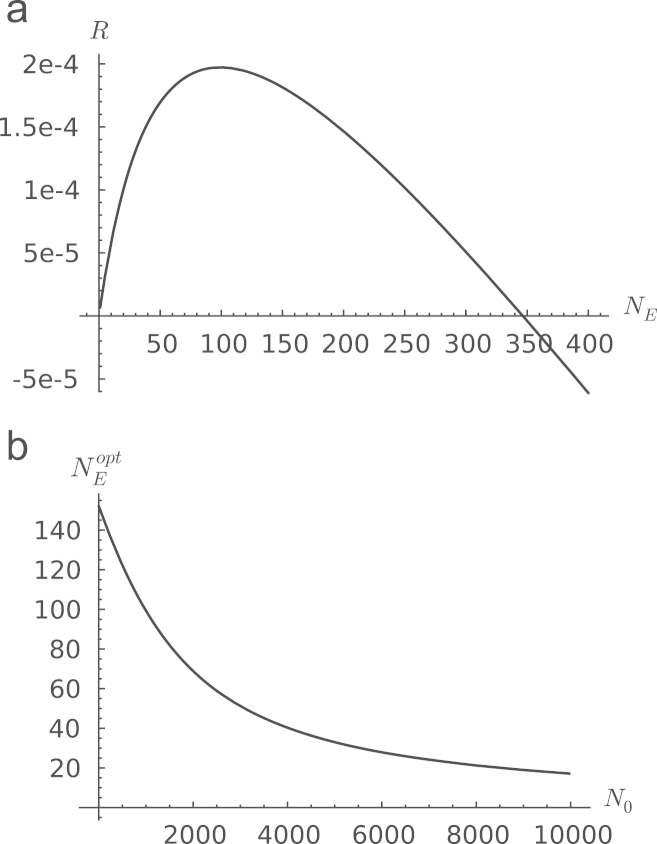
(a) Growth rate *R* as a function of *N*_*E*_ for $N_{0}=1000$, $N=5\times 10^{6}$, *L*=9, $T_{0}=1500\;s$; $\gamma =10^{-2}\;s^{-1}$; *f*=1. (b) Optimal repair enzyme number $N_{E}^{\mathrm{opt}}$ as a function of the initial number of damaged bases *N*_0_, for the same parameters.

Fig. 3a shows *R* as a function of *N*_*E*_, for a given number $N_{0}=1000$ of initial lesions, using parameters that are reasonable for *E. coli* (Husain et al., 1985; Ni et al., 2007). We find that *R* has a maximum at

(11) $$N_{E}^{\mathrm{opt}}=-\frac{N_{0}}{\gamma T_{0}}+\sqrt{{\left(\frac{N_{0}}{\gamma T_{0}}\right)}^{2}+\frac{N\;\ln\;2}{\mathrm{fL}\gamma T_{0}}}$$

$N_{E}^{\mathrm{opt}}=107$ for 1000 initial lesions. $N_{E}^{\mathrm{opt}}$ decreases with *N*_0_, and for $N_{0}=2000$ it is only 75.

### 4.2 Conclusion

Fig. 3b shows $N_{E}^{\mathrm{opt}}$ as a function of *N*_0_, from Eq. (11). Note that $N_{E}^{\mathrm{opt}}$ decreases monotonically with *N*_0_, and is never greater than $N_{E}^{\mathrm{opt}}(N_{0}=0)=160$, suggesting that repair enzymes in a cell should be kept at low numbers — although we point out that these results are not valid for very low *N*_0_. This conclusion is in accordance with current biological consensus that repair enzymes are expressed at low abundances, and the optimum values of *N*_*E*_ we found agree with the estimated numbers of repair enzymes that have been measured in cells (Myles and Sancar, 1989). Therefore, we suggest that evolution has constrained *N*_*E*_ to low values, so that the risks incurred during repair are optimally counter-balanced by the benefits provided by fast recovery.
